# Transcatheter Edge-to-Edge Mitral Valve Repair in Functional Mitral Regurgitation. Does it Pass Muster? Still Leaving Plenty to Be Desired

**DOI:** 10.21470/1678-9741-2022-0129

**Published:** 2022

**Authors:** Ovidio A García-Villarreal

**Affiliations:** 1 Mexican College of Cardiovascular and Thoracic Surgery, Mexico City, Mexico.

The usefulness of percutaneous approaches in structural heart disease could be limited if they have been defined in terms of often unrealistic scenarios. Thus, the long-term outcomes and consequences need to be painstakingly analyzed. Much attention needs to be paid to the global magnitude of this issue. One such example, is the transcatheter edge-to-edge mitral valve repair (TEER) and considerations of pivotal importance that arise from using this therapy to treat functional mitral regurgitation (FMR).

Traditionally, surgical treatment has been the best option for mitral valve (MV) repair. When comparing TEER to surgical MV repair, several salient details need to be considered. One of the main drawbacks of the edge-to-edge technique is that it has never been the first option for any surgical MV repair. Furthermore, the most important difference between the percutaneous technique and surgery is the absence of an annuloplasty prosthetic ring in the first option. Consequentially, this factor renders the percutaneous procedure only partially effective. It must be emphasized in the strongest terms that it is insufficient to consider TEER without an annuloplasty ring. We must also acknowledge the important role of the annuloplasty, by means of a prosthetic ring in every MV repair^[1,2]^. In fact, the lack of an annuloplasty ring is the most powerful predictor for failure after MV repair in the long term^[3,4]^. This rule is universal and, therefore, applies to any MV repair in the adult. Alfieri’s edge-to-edge technique that underpins the principle of TEER is no exception to this rule^[5-9]^. Nevertheless, it seems that all the implications of a ringless therapy such as TEER have not been completely addressed. Thus, we must define such limitations in the percutaneous approach for MV repair. Indeed, rules governing MV repair do not change just by shifting the approach. In fact, the percutaneous technique is constrained by the installation of a ringless TEER device. Due to TEER being a ringless therapy, dilatation of both commissures has been proposed as a possible explanation for the high occurrence of recurrent mitral regurgitation (MR) ≥ 2+ (23% at two years) observed in the Cardiovascular Outcomes Assessment of the MitraClip Percutaneous Therapy for Heart Failure Patients with Functional Mitral Regurgitation (COAPT) trial^[10]^.

The occurrence of FMR directly linked to coronary artery disease may constitute an ever-increasing potential indication for TEER. However, evidence supporting TEER in FMR is only limited to two controversial randomized controlled trials.

In the Multicentre Study of Percutaneous Mitral Valve Repair MitraClip Device in Patients with Severe Secondary Mitral Regurgitation (MITRA-FR) trial (in which primary funding was provided by the French Ministry of Health and Research National Program), TEER results were compared with medical treatment in patients with FMR. No statistically significant differences were observed between both groups for all-cause mortality (24.3% *vs.* 22.4%; hazard ratio [HR]: 1.11; 95% confidence interval [CI], 0.69 to 1.77) and rehospitalization for heart failure (HF) (48.7% *vs.* 47.4%; HR: 1.13; 95% CI, 0.81 to 1.56) at one year of follow-up^[11]^. At two years of follow-up, there was no significant difference for the composite of death for any cause and HF rehospitalization (63.8% *vs.* 67.1%; HR 1.01, 95% CI 0.77-1.34). Rehospitalization for HF alone did not show important difference between groups (55.9% *vs.* 61.8%; HR 0.97, 95% CI 0.72-1.30)^[12]^.

Contradictory results in favor of TEER were obtained in the COAPT trial, a study fully sponsored by the MitraClip™ device industry Abbott for TEER (MitraClipTM TMVr, Abbott, Santa Clara, USA) at 2 - 3- years of follow-up. Rehospitalization rate for HF was 35.8% for TEER and 67.9% in the control group (HR, 0.53; 95% CI, 0.40 to 0.70; *P*<0.001). In light of the above, important questions arise in respect of the best treatment for this special group of patients^[13]^.

Special efforts have been made in order to narrow the gap between the ideal and the real outcomes observed in COAPT and MITRA-FR trials, respectively. Theoretical explanation by means of the disproportionate/proportionate FMR concept has been proposed to provide a better understanding of the aforementioned data disparities^[14,15]^. The central concept is the effective regurgitant orifice area/end-diastolic left ventricular (LV) volume ratio. The selected cut-off value is 0.14 (with LV ejection fraction of 30% and regurgitant fraction of 50%, meaning severe FMR). Thus, two main types of FMR can be identified, namely, those with preserved LV volume and significant MR (disproportionate), and those with dilated LV volume and large MR (proportionate). The former is typical of chronic ischemic MV regurgitation with posterobasal deformation of the left ventricle due to chronic coronary artery disease. The latter is generally observed in dilated cardiomyopathies, regardless of the underlying etiology. Hence, at a first glance, disproportionate FMR cases entail better prognosis. However, when moving from theory into practice, all these tools have failed to obtain a reasonable explanation about the differences between trials until now. By using this concept, Lindenfeld et al.^[16]^ found no consistent differences between groups when comparing MITRA-FR-like *vs.* COAPT-like patients. Adamo et al.^[17]^ demonstrated that the relative risk of HF hospitalization and death was independent from the presence of disproportionate FMR^[18]^. In a study of 241 cases of TEER, Ooms et al.^[19]^ found no important difference for all-cause mortality and HF rehospitalization rates for disproportionate and proportionate FMR (30% *vs.* 37%, respectively). Hagendorff et al.^[18]^ have clearly emphasized that the disproportionate FMR can only be explained by means of conflicting data on the reported echocardiographic values, such as those observed in the COAPT trial.

It is important to clarify the distinction in the guideline-directed medical therapy (GDMT) between the two trials, in order to dispel the popular misconception about the similar equivalence between them. While post-procedural GDMT has been largely questioned in COAPT, intensive medical treatment was carried out and maintained in nearly 80% of cases in MITRA-FR. Thus, medical management was quite different in both trials, especially after randomization^[20]^. In the COAPT trial, angiotensin-converting enzyme inhibitors, angiotensin receptor blockers, or angiotensin/neprilysin receptor inhibitors were significantly underused in the medical therapy alone arm than in the TEER group throughout the whole trial period^[21]^. Taken together, these data tend to create a major distortion towards the most optimum balance in both arms of the COAPT. Hence, it could explain the wide different outcomes in both trials. In the same COAPT trial, within the group of patients whose MR did not improve 30 days after the start of medical treatment, 33% and 40% — at one year and two years, respectively — had significant improvement in reducing the severity of MR. This strongly questions the efficacy of the medical treatment used within the COAPT trial. Finally, if the pool of medications and doses used in COAPT (sacubitril/valsartan used in < 5% of cases, mineralocorticoid antagonist in 50%, and sodium-glucose cotransporter inhibitor in 0%) is compared with current treatment protocols, we can state that such medical therapies in the COAPT are, at present, inadequate and obsolete. Moreover, in stark contrast to what current clinical guidelines for the management of HF recommend as an aggressive medical treatment from the outset, reaching maximum doses as necessary^[22]^, the GDMT utilized in the COAPT trial leaves much to be desired.

Post-COAPT trial data considerations with respect to the absence of open, unbiased data transparency, alongside a lack of reproducibility in low-volume centers and the stringent patient selection criteria, make extrapolation to the majority of the population with FMR highly unlikely. Therefore, concern about the accuracy of all conclusions from the COAPT trial must be urged, and assiduous caution exercised when interpreting the results. Nonetheless, based on the COAPT results, the most recent European and American clinical guidelines for the management of valvular heart disease consider a Class IIa recommendation for TEER in FMR, in the absence of coronary artery disease. This recommendation is also applicable for cases of HF in stage D without an adequate response to GDMT^[23,24]^. The obvious conclusion that the COAPT results can be applied to most of the patients with FMR is, therefore, biased and unfounded. The author of this article considers that before accepting this recommendation, all available information should be revisited again in an impartial manner. In fact, the author has repeatedly suggested that this TEER recommendation for FMR should be a Class IIb recommendation, and limited to a very selective pool of patients^[25,26]^.

Much of the rationale for supporting TEER and downplaying MV repair surgery in FMR arises from research by Goldstein et al. and the Cardiothoracic Surgical Trials Network investigators, which demonstrated that the recurrent MR rate after MV restrictive annuloplasty was up to 58.8% at a two-year follow-up^[27]^. However, it is extremely important that this information be carefully considered. Firstly, specific echocardiographic parameters for MV repair in FMR were not included in this study. Significantly, systolic sphericity index > 0.7, LV end-systolic volume > 140 mL, LV diastolic diameter > 65 mm, LV systolic diameter > 51 mm, posterior papillary-fibrosa distance > 40 mm, interpapillary distance > 20 mm, coaptation depth > 1.1 mm, tenting area > 2.5 mm^2^, and posterior leaflet angle > 45° are considered as predictors of failure after restrictive annuloplasty in FMR^[28]^. The second fact is that no etiology-specific designed annuloplasty rings were utilized in the trial. The GeoForm annuloplasty ring (Edwards Lifesciences, Irvine, California, USA) and IMR ETlogix ring (CMA IMR ETlogix ring®; Edwards Lifesciences, Irvine, California, USA), instead of the classic Physio annuloplasty ring (Edwards Lifesciences, Irvine, California, USA), have demonstrated a lower rate of MV regurgitation recurrence after operation in FMR^[29,30]^.

The consequences of applying a therapy without anticipating long-term results can be devastating. In the CUTTING-EDGE study, 332 cases that underwent MR surgery after failed TEER were analyzed. Operative mortality was 16.6%, and 92.5% of cases ended with MV replacement^[31]^. Chikwe et al.^[32]^ reported 524 patients underwent MR surgery after failed TEER. Operative mortality was 10.2%, and 95% of these patients underwent MV replacement. The Transcatheter Mitral Valve Therapy STS/ACC/TVT Registry reed an occurrence of recurrent MR ≥ 3+ of 8.7% at just 30 days after TEER^[33]^. An analysis from data of the Heart Failure Network Rhineland registry showed MR ≥ 3+ of 9.8% at one year after TEER^[34]^, in stark contrast with the previously reported in the COAPT trial (5.3% at one year)^[33]^. Nevertheless, since many cases are not officially reported, there is vastly inadequate and insufficient information coming from the real world, regarding the need for reoperation after failed TEER.

In closing, durability of the TEER is currently unknown. A more complete technique for TEER should be a priority. Factors that govern the classic surgical principles for MV repair give genuine pause for thought. Why not begin to apply in TEER the same effective logic concerning surgical MV repair, in consideration of long-term durability and stringent patient selection by the most rigorous preoperative echocardiographic criteria?

Finally, every clinical trial must be governed by the highest standards of ethical research principles. It is imperative to have scientific trials that are unbiased at all levels of design and funding. In this way, clinical trial outcomes can contribute to raising the quality of human life throughout the world.
